# Multi-channel EEG recordings during 3,936 grasp and lift trials with varying weight and friction

**DOI:** 10.1038/sdata.2014.47

**Published:** 2014-11-25

**Authors:** Matthew D Luciw, Ewa Jarocka, Benoni B Edin

**Affiliations:** 1 IDSIA, Dalle Molle Institute for Artificial Intelligence, USI-SUPSI, CH-6928 Manno-Lugano, Switzerland; 2 Center for Computational Neuroscience and Neural Technology, Boston University, Boston, Massachusetts 02215, USA; 3 Physiology Section, Department of Integrative Medical Biology, Umeå University, S-90187 Umeå, Sweden; 4 Department of Kinesiology, Faculty of Physiotherapy, University School of Physical Education, Wroclaw 51-612, Poland

## Abstract

WAY-EEG-GAL is a dataset designed to allow critical tests of techniques to decode sensation, intention, and action from scalp EEG recordings in humans who perform a grasp-and-lift task. Twelve participants performed lifting series in which the object’s weight (165, 330, or 660 g), surface friction (sandpaper, suede, or silk surface), or both, were changed unpredictably between trials, thus enforcing changes in fingertip force coordination. In each of a total of 3,936 trials, the participant was cued to reach for the object, grasp it with the thumb and index finger, lift it and hold it for a couple of seconds, put it back on the support surface, release it, and, lastly, to return the hand to a designated rest position. We recorded EEG (32 channels), EMG (five arm and hand muscles), the 3D position of both the hand and object, and force/torque at both contact plates. For each trial we provide 16 event times (e.g., ‘object lift-off’) and 18 measures that characterize the behaviour (e.g., ‘peak grip force’).

## Background & Summary

The idea of extracting signals related to object manipulation from EEG recordings in humans seems reasonable given that even basic motor tasks engage large parts of the human cortex^1^. It is, however, not known how much information can actually be decoded from EEG. Specifically, it is unclear to what extent it is possible to extract signals *useful* for monitoring and control of manipulation tasks, for instance, to control an upper limb prosthetic device to generate a power grasp or a pinch grasp involving the thumb and index finger. While successful EEG decoding of reaching trajectories has been reported^2^, this claim is controversial^3^.

We present a dataset that allows critical evaluations of the utility of EEG signals for prosthetic control of object manipulation. It is based on an established and prototypical paradigm to study precision grasp-and-lift (GAL) of an object, introduced in the early 1980 s by Johansson & Westling^4–6^, and subsequently used in thousands of studies.

The correct completion of the GAL task depends on multimodal sensory activity correlated with specific *events* such as object contact, lift-off, and replacement. This control policy, in which feedforward control routines operate between sensed discrete events, is known as the *Discrete Event Sensory Control* policy (DESC; refs 7,^8^, ^9^). As these events are crucially important for effective GAL, if they cannot be predicted from the EEG signal, than the EEG signal is of limited use for BCI control of robot hand manipulation.

We collected data from twelve participants in the new dataset WAY-EEG-GAL (WAY: Wearable interfaces for hAnd function recoverY, the funding European project), which contains a total of 3,936 (=12 · 328) grasp and lift trials. The participant’s task in each trial was to reach for a small object, grasp it using their index finger and thumb, and lift it a few centimetres up in the air, hold it stably for a couple of seconds, and then replace and release the object. The beginning of the reach and the lowering of the object was cued by an LED, otherwise the pace of the task was up to the participant. During all trials, we recorded 32 channels of EEG, 5 channels of EMG from the shoulder, forearm, and hand muscles, the position of the arm, thumb and index finger and the object, and the forces applied to the object by the precision grip. We defined 16 behaviourally relevant events and extracted them for every trial. These event times are available along with the scripts used to generate them and the raw data.

In all series, the object’s properties were several times changed in a manner that was unpredictable to the participant with respect to weight (165, 330, or 660 g), contact surface (sandpaper, suede, or silk), or both. Such changes are known to induce specific modifications to the required muscle coordination. For example, both *grip force* and *lift force* must increase when the object’s weight increases, whereas only the *grip force* must increase when the object’s weight is unchanged but the surface friction is decreased. We confirmed that all participants adjusted their fingertip forces according to the object’s properties.

The size and richness of this dataset enables investigations of the information content of EEG during dextrous manipulation; for instance, can EEG be used to identify

- the intention to reach and grasp?
- the hand positions and velocities?
- the onset of the load phase, i.e., the participant’s intention to apply lifting forces?
- when an object is replaced on a support for subsequent release?
- that the properties of the object have unexpectedly changed?

In short, are EEG signals reliable for the control of prosthetic devices? Combining the well-defined GAL paradigm with EEG recordings allows investigations into the information content of EEG and has the potential to lead to new EEG-based techniques for prosthetic device control.

Data and scripts have been made available under the terms of *Attribution 4.0 International Creative Commons License* (http://creativecommons.org/licenses/by/4.0/).

## Methods

### Participants

An ad calling for participation was posted at Umeå University, summarizing basic information about the study and promising 100 SEK per hour for at least two hours. Among those who responded, only right-handed individuals were selected as participants (*n*=12, 8 females, age 19–35) and they signed a consent form (included within Supplementary File 1—*Information.pdf*) in accordance with the Declaration of Helsinki. The experimental procedures were approved by the Ethical Committee at Umeå University. The participants were told that ‘the aim is to study how the brain and muscles are coordinated when handling an object’.

### Sensors

**Figure 1:**
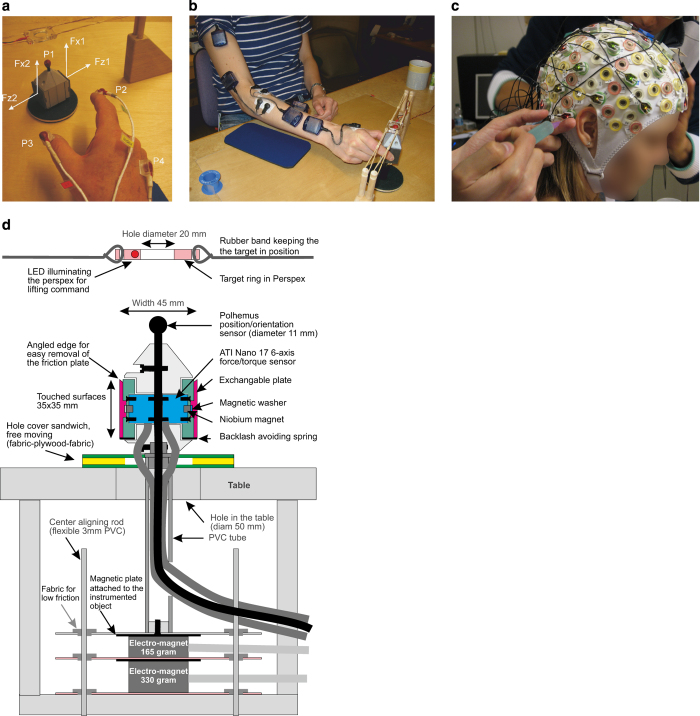
*Methods*. (**a**) Force and position sensors. F1-F2 correspond to force/torque sensors (ATI Nano), with x corresponding to lift force and z to grip force. P1-P4 correspond to 3D position sensors (Polhemus FASTRAK) attached to the object (P1), the index finger (P2), the thumb (P3) and the wrist (P4). (**b**) EMG sensor placement: 1-anterior deltoid, 2-brachioradialis, 3-flexor digitorum, 4-common extensor digitorum, 5-first dorsal interosseus. (**c**) EEG sensor (ActiCap), recording from 32 electrodes. (**d**) *Test object.* The object to be grasped was visible on top of the table (cf, panel a) while the rest was hidden from view. The distance between the two contact surfaces (each 35×35 mm) measured 45 mm and they were secured to the object by niobium magnets. The touched surface could easily be replaced. The force applied to each contact plate was measured with mechanically isolated ATI Nano 17 6-axis force/torque sensors. The weight of the object including its magnetic plate was 165 g and could be increased to 330 or 660 g by controlling two electromagnets at the bottom. A set of flexible PVC rods provided low-friction alignment of the object on the table. Above the object was a rectangle in perspex with a centre hole (Ø20 mm). The start of the trial was signalled when the Perspex rectangle was illuminated by a LED. On top of the object was a Polhemus sensor mounted to record the position and orientation of the object.

Four types of carefully placed sensors recorded kinematics, forces, muscle activations, and brain activity. Four 3D position sensors (labelled P1-P4 in Figure 1a; FASTRAK, Polhemus Inc, USA; links to equipment information are provided in Supplementary File 1) recorded the position (XYZ Cartesian coordinates) and orientation (azimuth, elevation, and roll) of the object, the index finger, the thumb and the wrist. On the sides of the object there were two surface contact plates each coupled to a force transducer that recorded 3 force and 3 torque channels (ATI F/T 17; Figure 1d). The five electromyography (EMG) sensors (Figure 1b), were placed on pertinent right arm muscles, viz., the anterior deltoid, brachioradial, flexor digitorum, common extensor digitorum, and the first dorsal interosseus muscles (Figure 1b). The EEG cap (Figure 1c; ActiCap) recorded from 32 electrodes in a standard configuration (an image file showing the electrode locations and a data file with the channel coordinates are available in Supplementary File 2—*Utilities.zip*).

### Data acquisition

The EEG cap was used in conjunction with a BrainAmp EEG signal amplifier. BrainAmp sampled at 5 kHz and band-pass filtered each channel from 0.016–1,000 Hz. The amplifier software VisionRecorder digitized and filtered the raw EEG data, and passed it to BCI2000^10^ for data storage. A target sampling rate of 500 Hz was set in the amplifier software, which used an adapted low-pass filter to prevent aliasing.

All other signals were sampled using SC/ZOOM (developed at Department of Integrative Medical Biology, Umeå University). The EMG signals were sampled at 4 kHz, and all others at 500 Hz. In addition to the kinetic and kinematic sensors, we recorded the object’s state, i.e., the prevailing contact surface (sandpaper, suede, silk) and weight (165 g, 330 g, 660 g), the state of the LED that indicated to the participant to start and terminate a trial (Figure 1b,d), and the state of the LED that showed the researcher to change contact surfaces.

To enable secure synchronization between SC/ZOOM and the EEG recording system, SC/ZOOM generated a continuous random signal that jumped between 0 and 1 at ~4 Hz which was recorded in both systems. By analysing the lags in the cross-correlation of the two respective sync channels, the EEG signals and the SC/ZOOM signals could be synchronized with an error ≤2 ms.

### The object

The object to be grasped and lifted was only partially visible to participants (Figure 1b,d). When the object was lifted, a PVC tube (Ø50 mm) that contained the cables from the force transducers and the Polhemus sensors also became visible.

The object’s weight and contact surface plates could be changed between trials without changing the object’s visual appearance. Changing the surface required researcher intervention. The surfaces were attached to the object by niobium magnets and could easily be replaced. Changing the weight was automated, by activating one or both electromagnets at the bottom of the device (Figure 1d; Supplementary File 3—*Weight Changes.avi* shows a cutaway view of the weight changing mechanism under the table, for two transitions, from 165 to 330 to 660 g). Several flexible and low-friction PVC rods under the table helped in centre-aligning the object during lifting tasks.

A translucent Perspex rectangle with a centre hole (Ø20 mm) was suspended by rubber bands above the object (Figure 1b,d). A LED mounted in the rectangle could be turned on and off: each trial commenced when the rectangle was illuminated and ended (i.e., the object was to be replaced on the table) when the LED was turned off. The participants were asked to lift the object such that the Polhemus sensor (labelled P1 in Figure 1a) was positioned at the centre of the hole of the Perspex rectangle.

### Preparation

The instruction documents used by the researchers and given to the participant before the experiment, respectively, are available in Supplementary File 1. The per-lift instructions to the participants were:

*Sit close to the table, relax your shoulder and place your upper arm next to your body. The elbow joint shall be higher than the wrist. During performance of the task, the forearm shall not touch the table. Your left arm should rest close to your waist. The red light is the signal to reach out and lift the object. Grasp the object with your thumb and index finger, in the middle of the grey surface and lift the object about 5 cm from the table. You should lift the object into the circle and hold it there until the red light turns off. Place the object on the table and place your arm next to your body. You shall rest your hand on the ‘blue surface’—relax your shoulder.*

Asking the participants to position the small red sphere on the top of the object in the opening at the centre of the illuminated rectangle provided an (albeit trivial) task objective, when performing the potentially boring task of lifting the object hundreds of times. They were given sound-masking earplugs to wear during the task.

Each experiment was carefully monitored and controlled: One experimenter controlled SC/ZOOM that recorded all non-EEG signals and generated the sync signal, was in charge of changing the surfaces on the object, and made sure that the participant followed the protocol (e.g., by returning the hand to the blue surface after a trial). A second experimenter was in charge of the EEG signals and their recording, which started after he verified the sync signal’s appearance.

There were three alternate surface pairs, one for each surface type. During series involving surface change, the researcher replaced the surfaces on the object between every trial, sometimes to the same surface type and the stand with surface plates were kept out of the participant’s view. To further eliminate any useful predictive cues, the experimenter always made the same movements and the plates were constructed to be visually practically indistinguishable.

The researcher knew which surface to select based on the lighting pattern of LEDs which were controlled by SC/ZOOM. After replacing the surface, the experimenter pressed a button, which caused SC/ZOOM to generate a random time interval between 0 and 2 s, after which the participant’s LED turned on. During trials without surface change, the light would automatically turn on once the participant digits had been at least 15 cm away from the object for 1–3 s. The participant LED turned off automatically after the object had been in the circle for 2 s.

A video of an example trial is included as Supplementary File 4—*Example Trial.avi*. The participant waits with the arm resting on the blue surface while the assistant changes the contact plate. The assistant gets out of the way. The participant watches the LED. It turns on. The participant reaches for the object and grips it with forefinger and thumb. The participant lifts the object, holding the red sphere steady within the circle. The LED turns off. The participant lowers and releases the object in its resting place. The participant retracts the arm, back onto the blue surface and the trial is over.

### Series

Each participant performed 5 different types of experimental series. The *practice series* involved repeated lifting with the object at 330 g to familiarize the participant with the task (the practice series was not included in the extracted data).

The *weight series* involved 34 lifts with 12 unpredictable weight changes (between 165, 330 and 660 g). Six different weight series schedules were constructed so that the same weight was repeated 1–4 times and then changed. The *friction* or *surface series* involved 34 lifts with variable surface friction (sandpaper, suede or silk). Six different series schedules were constructed using the same logic as for the weight series. All sequences and changes were balanced across the constructed series. During all weight series, the contact surfaces were sandpaper and during all surface series, the object’s weight was 330 g. The *mixed series* had 28 lifts including 11 lifts with an unexpected change in the object’s weight (to 165 or 330 g; *n*=4), contact surface (to sandpaper or silk; *n*=3), or both (*n*=4).

The final type of series was the *friction estimation* series. It included up to 34 trials where the participant held the 330 g object in the air and slowly spaced the digits until slip occurred at one of the digits. The friction estimation series did not include EEG recordings.

The data available on *figshare* (Data Citation 1) includes 10 experimental series from each participant: 6 weight series, 2 friction series and 2 mixed series.

The series schedule is included in Supplementary File 1. A complete account of the series including current and previous weights and surfaces for every trial is provided for each participant in the *P.AllLifts* structure (described below in the section Data Records).

### Data processing

Raw data from SC/ZOOM and BCI2000 was imported to MATLAB to prepare the data records. The maximum of the cross-correlation, applied to the two sync signals (using the function *xcorr* with a maximum possible time lag of 5,000 samples) indicated the time lag shift, to sync the signals. We also removed unneeded or extra samples at the beginning and end of each series. The only pre-processing done to the data was to remove the mean from the EMG signals. No artefact rejection (blinking, eye movements, etc) was applied to the EEG signals.

Three types of data structures were prepared. The per-series kinematic, kinetic and neurophysiological data were stored in two complementary types of structured data files. The first structure—*holistic*—simply includes the raw data for each lifting series. The second type of structure—*windowed*—organizes the series into temporally segmented windows around each individual lift. Each window starts exactly two seconds before the LED that cued the participant turned on, and ends three seconds after this LED turned off.

Derived signals based on *grip and load force*s were calculated and added to the windowed structures. The total grip force was calculated as (Fz1+Fz2)/2, while the load force was calculated as Fx1+Fx2 (Figure 1a). Per-digit and total forces were calculated, along with the grip force: load force ratios. The ratios were only calculated when the absolute load force was >0.1 N.

A third data structure contains high-level information about each lift, such as the object’s surface and weight properties, these properties for the previous lift in the series, a set of *extracted event* times, and a set of measures that characterize the behaviour.

### Event extraction

**Figure 2:**
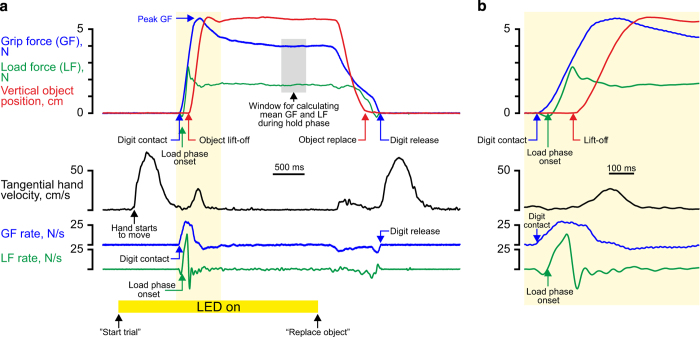
*Sample trial*. (**a**) Complete trial from just before the onset (indicated by the illumination of the Perspex plate above the object) until that object weighing 165 g was replaced and the hand returned to the starting position. Arrows labelled *Digit contact*, *Load phase onset*, etc, mark some of many ‘events’ extracted from all single trials (*cf*. Table 1). (**b**) The window marked by a yellow rectangle in (**a**) shown on an expanded time scale.

Events structure the lift sequence, as seen in Figure 2. These events include the LED turning on and off, the index finger and thumb first making contact with the object, the onset of the load phase, lift-off, object placement on its support, object release, and the hand returning to the blue surface. The time of these (and more) events, and other related information (such as the duration of the various *phases*, between certain events), was extracted for every lifting trial and included as part of this dataset. 43 pieces of per-trial information are stored. The methods of computation of most of these components are easily inferred from the short descriptions provided (the script file *WEEG_FindEvents.m*, included in Supplementary File 2, provides all details). For all events, extensive inspections of both recorded time-series and histograms of the identified events confirmed that the algorithms worked as intended.

To identify many events a combination of 1st and 2nd time derivatives of the pertinent signals were employed. Before computing these derivatives, all signals were subjected to Savitzky-Golay filtering. For instance, to obtain the derivatives of the signal **X**, the following MATLAB code was used:


dX = sgolayfilt ([0; diff (X)], 3, SGF_WIN_)/dt;
ddX= sgolayfilt ([0; diff (dX)], 3, SGF_WIN_)/dt;


with SGF_WIN=31 and dt=1/sampling rate.

To find the time of onset of the hand movement, the tangential velocity was calculated:


HandVel = sqrt (dX. ˆ2 + dY.ˆ2 + dZ.ˆ2); 


The moment when HandVel reached 1 cm/s was then defined as the onset of movement.

To identify the moment of touch, we used the moment when the normal force had increased above 4 times the standard deviation of the normal force during hand movements.

A more complex algorithm was required to identify the onset of the load phase. Often participants moved along an upward convex trajectory towards the object and therefore tended to apply a downward tangential force when they initially grasped the object, i.e., they generated an initial ‘negative load force’ (e.g., Figure 2b). To resolve this, the moment of the zero crossing of the 2nd derivative of the load force immediately before the LF had reached 0.2 N was found and this could be used whether the initial LF was positive or negative.

## Data Records

Supplementary File 1 contains all the series schedules, listing for each participant the sequence of actual experimental series and in what order they were stored in the data structures. The PDF also includes demographic information and notes about each participant.

The *HS_PX_SY.mat* file (where *X* is participant number and *Y* is series number), contains a structure with all data in a single lifting series. The *WS_PX_SY.mat* files contains a structure with the data organized in windows around every single lift, to allow easy extraction of single trials. The *PX_AllLifts.mat* file contains a structure *P* with information about every lift performed by each participant *X*, such as the times at which specific events occurred.

For each of the 12 participants, a single *P* structure is provided, and one *HS* structure and one *WS* structure are provided *for each series*. However, for a single weight series per participant, the non-EEG information was excluded, and is kept secret for a later competition. The total size of all MATLAB data structures, for all participants, stands at ~15 GB.

### HS_P1_S1.mat—HS_P12_S9.mat (108 files)

Each file contains all data in a single lifting series, in continuous format. For example, HS_P3_S2.mat contains the data for the Series 2 of Participant #3. Basic ID information is in the top level of the structure. *hs.name* gives the participant initials, and *hs.participantnum* and *hs.seriesnumber* give the participants data record number and the number of the series. Each of *hs.emg, hs.eeg, hs.kin, hs.env,* and *hs.misc* are substructures with the following fields: .names, .samplingrate, and .sig. Each .sig is a matrix of dimension #samples x #channels and contains the actual data. An identifier for each column of these matrices is found in *names*. The five matrices of each holistic structure are *eeg*, containing 32 EEG signals, *emg* (5 EMG signals), *kin* (24 position sensor signals and 12 force plate signals), *env* (the surface and weight signals), and *misc* (the remaining recorded signals—the surface LED signal, the participant LED signal, the button pressed by the researcher, the magnet signal, and two temperature signals).

### HS_P1_ST.mat—HS_P12_ST.mat (12 files)

Each of these files contains the *eeg* matrix, but not *emg*, *kin*, *env*, or *misc*.

### WS_P1_S1.mat—WS_P12_S9.mat (108 files)

Each file contains all data in a single lifting series, in windowed format. For example, WS_P2_S3.mat contains the data from the 3rd series of Participant 2. These files contain:


ws.id Participant’s initials
ws.participantnum Participant’s number
ws.seriesnumber Series number
ws.win(trial#) A structure for each single lifting trial containing the following fields:
.eeg samples×32 channels (channel names found in ws.names)
.kin samples×45 channels (channel names found in ws.names)
.emg samples×5 channels (channel names found in ws.names)
.eeg_t samples×1 giving time of each row in.eeg and.kin
.emg_t samples×1 giving time of each row in.emg
.trial_start_time absolute starting time (=StartTime in AllLifts,)
.LEDon time of LED onset (=LEDOn in AllLifts)
.LEDoff time of LED offset (=LEDOff in AllLifts)
.weight integer corresponding to weight (CurW in AllLifts)
.weight_id text representing the weight (e.g., ‘330 g’)
.surf integer corresponding to surface (CurS in AllLifts)
.surf_id text representing the surface (e.g., ‘silk’)
.weight_prev integer corresponding to weight in the previous trial
.weight_prev_id text representing the weight in previous trial
.surf_prev integer corresponding to the surface in the previous trial
.surf_prev_id text representing the surface in previous trial
ws.names Contains names for name fields in the data structures of ws.win
.eeg name of columns in the ws.win(n).eeg matrix
.kin name of columns in the ws.win(n).kin matrix
.emg name of columns in the ws.win(n).emg matrix


Nine derived signals are included in *ws.kin*,


Column 37 index finger load force
Column 38 thumb load force
Column 39 total load force
Column 40 index finger grip force
Column 41 thumb grip force
Column 42 averaged grip force
Column 43 index finger grip force/load force ratio
Column 44 thumb grip force/load force ratio
Column 45 total grip force/load force ratio 


### P1_AllLifts.mat—P12_AllLifts.mat (12 files)

The matrix *P.AllLifts* contains one row for each recorded lifting trial and 43 columns that each represents a variable pertaining to single trials. The names of the columns in *P.AllLifts* can be found in *P.ColNames*. Table 1 describes the contents. Note that all times (except StartTime) are relative to the *window* start.

### Data repository

Data are available at *figshare* (Data Citation 1).

## Technical Validation

### EEG data

During data acquisition, unexpected artefacts in the EEG signals (e.g., 50 Hz electrical noise) and the impedance of each EEG electrode were continually monitored. One experimental series was aborted when noise was evident because of technical problems, and that series was restarted once the problem had been fixed.

**Figure 3:**
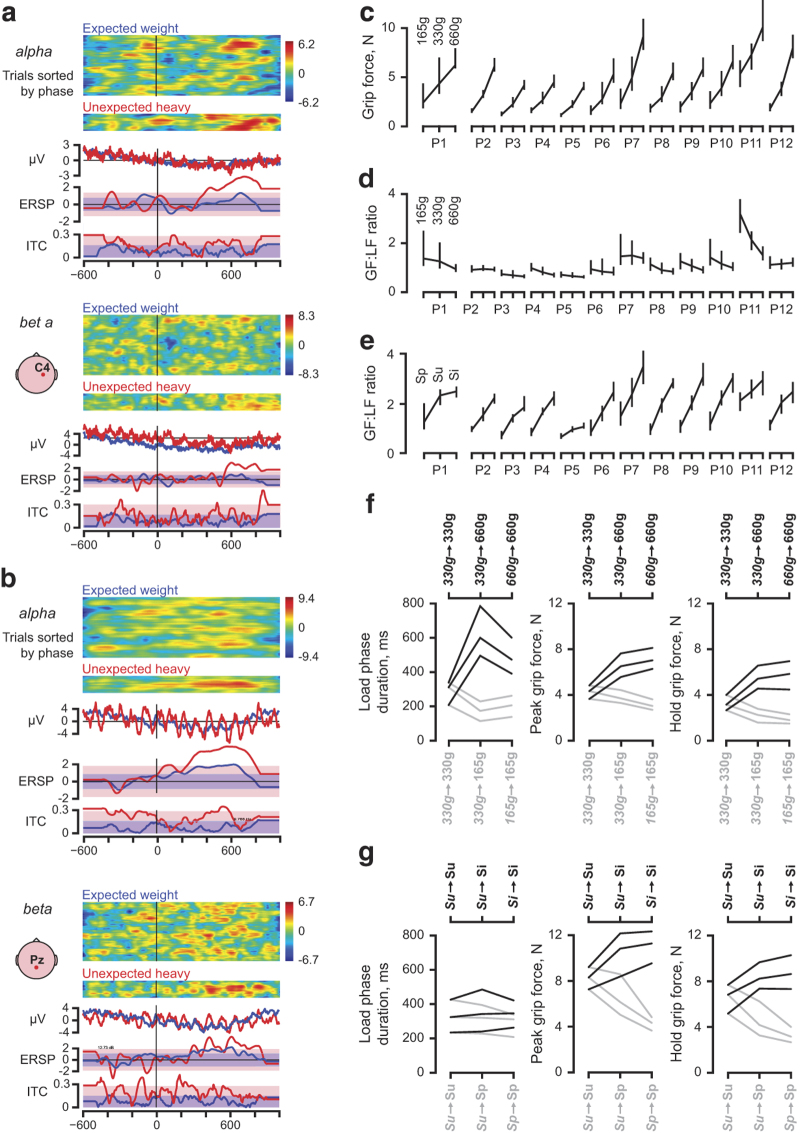
*Data validation*. (**a**,**b**) For channels C4 and Pz (shown in insets) recorded in Participant 3, trials when the object's weight was the same as in the previous trial (*Expected* weight, *n*=105; blue lines) and unexpectedly heavy (*n*=30; red lines) were contrasted using EEGLab^11^. The panels show from top, the power in the alpha and beta bands after sorting the trials by phase at the peak frequency, the average EEG amplitudes, the ERSPs and the ITCs. The colored patches represent 95% confidence intervals. The earliest moment this participant on average could have detected an increased object weight was ~200 ms after object contact (i.e., time zero). (**c**) All participants adapted their grip force to the object’s weight, i.e., 165, 330 or 660 g in series with sandpaper surfaces. The different weights thus invoked markedly different fingertip forces in all participants. (**d**) The grip:load force ratio was the same or declined across the three object weights in all participants, i.e., the force coordination was roughly the same irrespective of the object’s weight. (**e**) In series with the same object weight (330 g) but with contact plates covered with sandpaper, suede or silk, the grip:load force ratio increased with decreasing friction, i.e., in all participants the three contact plates offered different object-fingertip friction and all participants adapted to the prevailing friction. (**f**,**g**) When the weight (**f**) or the contact surfaces (**g**) was unexpectedly changed between trials, there was a marked change in the load force duration, in the peak grip force and the hold grip force (e.g., all increased when the object had an unexpected increased weight or decreased friction). Data aggregated across all participants. The lines represent the median and the 1st and 3rd quartile, black lines increased weight (**f**) and increased slipperiness (**g**) and gray lines decreased weight and slipperiness, respectively, as indicated on the top and bottom axes.

That recorded EEG was confirmed to change with behavioural conditions as illustrated for Participant 3 in Figure 3a,b. The recordings were synchronized at the moment when both digits had made contact (*tBothDigitTouch*, Table 1). With the help of EEGLab^11^, trials with the same weight as in the previous trial were contrasted with those with an unexpectedly higher weight. This participant showed a median time of 166 ms (interquartile range of 90 ms) from object contact to object liftoff, i.e., *tLiftOff*‒*tBothDigitTouch*, when the object's weight was 165 or 330 g, i.e., the earliest moment the unexpected weight could have been detected by the participant was after ~200 ms. Indeed, the EEG changed after this as exemplified for the Pz and C4 channels in power of the alpha (8–13 Hz) and beta (15–25 Hz) bands, the ERSP (*event-related spectral perturbation*) and the ITC (*inter-trial coherence* or *event-related phase-locking*).

### EMG data

The quality of each EMG signal, i.e., the amplitude when the corresponding target muscle was activated, was assessed before the lifting trials commenced and continually during the experiments by means of online monitors. When signals deteriorated during an experimental run, notes about this were made (detailed in Supplementary File 1).

### Kinematic and kinetic data

The setup was designed to minimize any interference with the 3D position recording system, that is, wood or plastic materials were used whenever possible in the object and the table. The measurement rms errors within the work space were confirmed to be ≤0.1 mm and ≤0.2° for the position and angular readings, respectively. Prior to the experiments, all sensors in the test objects were carefully calibrated.

### Behavioural validation

Figure 3c–e demonstrates that all participants adjusted the force coordination to the prevailing weight and friction. Moreover, and importantly, an unpredictable change of the object’s weight or surface material, resulted in marked changes but these effects were largely eliminated already in the subsequent lift, i.e., most of the adaptation took place in single trials (Figure 3f,g). The participants’ behaviour thus replicates the major findings in previous studies^4–6^ and the data show that significant behavioural effects were indeed evident in the recorded trials as a consequence of the object’s properties.

## Usage Notes

All data files (archived, per-participant, in zip format) are available from *figshare* (Data Citation 1). Several potentially useful MATLAB scripts are archived in Supplementary File 2—*Utilities.zip*. In this archive file, *Usage.txt* provides short instructions about using the code. These scripts are additionally made available through GitHub, at https://github.com/luciw/way-eeg-gal-utilities.

We provide:

**WEEG_GetEventsInHS.m** returns the times of various events within the series, instead of within the windowed trials. One can select the participant and a particular series type—Weight, Friction, Mixed, or All.

**WEEG_PlotLifts.m** enables a per-window visualization of a participant’s activities. One can select the participants and series to plot. Each subplot shows a different trial and displays three signals: the grip force, the load forces and the hand velocity. Seven events are indicated by dotted vertical lines. The events shown are the time of: LEDon, when the hand starts moving, first contact, liftoff, LEDoff, the object is placed down, and object release. Above each subplot, the weight and surface type are indicated.

**WEEG_PlotStats.m** displays histograms indicating, per-participant, the time of index finger contact relative to thumb contact, the duration of the preload phase, and the duration of the load phase. The load phase duration is broken into 9 subplots, shown in the 3×3 grid. The current weight is shown on the y-axis, while the weight in the previous trials is shown on the x-axis.

**WEEG_FindEvents.m** is the script used to determine event timings and lift characterizations, and was used to generate the **P.AllLifts** structure.

The MATLAB data files and scripts described above can be loaded and run with Octave 3.8 or higher (http://www.gnu.org/software/octave/).

The open-source MATLAB software EEGLab^11^ can be used to assist in processing the EEG signals. We provide two scripts for importing the data to EEGLab. **WEEG_MakeEEGLABDataset.m** and **WEEG_MakeAllEEGLABDatasets.m** convert the EEG and event data for all series into EEGLAB 'sets', for one participant and for all participants, respectively. The file **chanlabels_32channel.xyz** (in Supplementary File 2) is used to localize the electrode positions in EEGLab, for topographic plots. **WEEG_HowToGenerateERSP.txt** describes how to use EEGLab to detect event-related spectral perturbation (ERSP) in the WAY-EEG-GAL data.

### Characterization of events

When exploring the WAY-EEG-GAL dataset it may be useful to consider that some events are primarily preceded by and others are followed by central nervous system activity. For instance, the reaching phase is reasonably preceded by brain activity that may be reflected in the EEG *prior* to the initiation of the hand movement, while touching the object gives rise to sensory inputs that may be reflected in the EEG *after* the event.

## Additional information

**How to cite this article:** Luciw, M. D. *et al.* Multi-channel EEG recordings during 3,936 grasp and lift trials with varying weight and friction. *Sci. Data* 1:140047 doi: 10.1038/sdata.2014.47 (2014).

## Supplementary Material

Supplementary File 1—Information.pdf

Supplementary File 2—Utilities.zip

Supplementary File 3—Weight Changes.avi

Supplementary File 4—Example Trial.avi.

## Figures and Tables

**Table 1:** P.AllLifts—matrix describing all single trials.

**Column**	**Variable**	**Unit**	**Description**
1	Part	integer	Participant number
2	Run	integer	Series number
3	Lift	integer	Sequential trial within series
4	CurW	integer	Current weight—[1=165 g, 2=330 g, 4=660 g]
5	CurS	integer	Current surface—[1=sandpaper, 2=suede, 3=silk]
6	PrevW	integer	Weight in previous Lift—[1=165 g, 2=330 g, 4=660 g]
7	PrevS	integer	Surface in previous Lift—[1=sandpaper, 2=suede, 3=silk]
8	StartTime	seconds	Start time relative to start of series.
9	LEDOn	seconds	Time when the LED in the Perspex plate was turned on; this the signal to the participant to commence a Lift (always 2 )
10	LEDOff	seconds	Time when the LED in the Perspex plate was turned off; this was the signal to the participant to replace the object
11	BlockType	integer	Type of Series—[1=Weight series; 2=Friction series; 3=Mixed weight and friction series]
12	tIndTouch	seconds	Time when the index finger touched the object
13	tThumbTouch	seconds	Time when the thumb touched the object
14	tFirstDigitTouch	seconds	Time when the first digit touched the object
15	tBothDigitTouch	seconds	Time when both digits have touched the object
16	tIndStartLoadPhase	seconds	Time when the index finger start to apply load force
17	tThuStartLoadPhase	seconds	Time when the thumb finger start to apply load force
18	tBothStartLoadPhase	seconds	Time when both digits have started to apply load force
19	tLiftOff	seconds	Time when the object lifted off from the support
20	tReplace	seconds	Time when the object was replaced on the support
21	tIndRelease	seconds	Time when the index finger released the object
22	tThuRelease	seconds	Time when the thumb released the object
23	tBothReleased	seconds	Time when both digits have released the object
24	GF_Max	N	Maximum grip force (mean of the maximum GF applied by the index finger and the thumb)
25	LF_Max	N	Maximum load force (sum of the maximum LF applied by the index finger and the thumb)
26	dGF_Max	N/s	Maximum GF rate
27	dLF_Max	N/s	Maximum LF rate
28	tGF_Max	seconds	Time when the maximum GF occurred
29	tLF_Max	seconds	Time when the maximum LF occurred
30	tdGF_Max	seconds	Time when the maximum GF rate occurred
31	tdLF_Max	seconds	Time when the maximum LF rate occurred
32	GF_hold	N	Mean GF in a 200 ms time window starting 300 ms before LEDOff
33	LF_hold	N	Mean LF in a 200 ms time window starting 300 ms before LEDOff
34	tHandStart	seconds	Time when the hand starts to move (after LEDOn)
35	tHandStop	seconds	Time when the hand stops (returned to blue area)
36	tPeakVelHandReach	seconds	Time when the tangential hand velocity reaches its maximum during the reaching phase
37	tPeakVelHandRetract	seconds	Time when the tangential hand velocity reaches its maximum during the retraction phase
38	GripAparture_Max	cm	Maximum grip aperture (MGA) during the reaching movement
39	tGripAparture_Max	seconds	Time of MGA
40	Dur_Reach	seconds	Duration of the reaching phase (from start of hand movement to initial object touch)
41	Dur_Preload	seconds	Duration of the preload phase, i.e., from digit contact until LF application commenced
42	Dur_LoadPhase	seconds	Duration of the load phase, i.e., from LF was applied until object lift-off
43	Dur_Release	seconds	Duration of the release phase, i.e., from moment the object was replaced on the table (tReplace) until both digits had released the object
