# Supplementary material for: Multi-channel EEG recordings during 3,936 grasp and lift trials with varying weight and friction
Source: Sci Data. 2014 Nov 25;1:140047. doi: 10.1038/sdata.2014.47 (PMC4365902; doi:10.1038/sdata.2014.47)
Supplement: Supplementary File 1—Information.pdf [file sdata201447-s1.pdf]

Supplementary information

Table of contents

Consent form (in Swedish) ____ 2

Links to suppliers of equipment used __________________________________ 4

Series schedule for each participant _____________________________________5

Information about participants _______________________________________ 6

Check-list for instructions to participants _________________________________7

UMEÅ UNIVERSITET
Institutionen för Integrativ medicinsk biologi

Informerat samtycke för deltagande i forskningsprojekt gällande Lift and Reach.

Syfte.
Projektet syftar till att studera hur hjärna och muskler koordineras vid hantering av ett föremål.

Hur går studien till?
Du kommer att få utföra en uppgift som går ut på att lyfta ett föremål från ett bord, ca 5 cm från bordsskivan och ställa ned föremålet på bordet igen. Uppgiften upprepas många gånger.
Du kommer att använda tumme och pekfinger på höger hand när du lyfter föremålet.

Handens, pekfingrets och tummens position registreras genom att sensorer placeras på dessa ställen (Sensorerna sätts fast med dubbelhäftande tejp).
Aktiviteten i vissa muskler registreras genom att vi placerar ytelektroder på huden. Ytelektroderna sätts fast med tejp.
Aktiviteten i hjärnan registreras genom att en hätta med elektroder placeras på ditt huvud. Hättan knyts under hakan. För att få god kontakt mellan elektroden och huvudet används elektrodgel (detta gör att du blir kladdig i håret).

Tid och ersättning.
Varje tillfälle pågår i ca 3½ timmar. Ersättning utgår med 100 kr för varje påbörjad timme.

Vilka är riskerna?
Det finns inga risker med att delta i denna studie.

Hantering av data och sekretess.
Dina svar och dina resultat kommer att behandlas konfidentiellt så att inte obehöriga kan ta del av dem. Dina resultat kommer inte att presenteras enskilt utan sammanställs anonymt med alla andra deltagares. Det kommer inte att gå att identifiera dig som person när resultaten sammanställs. Personuppgifter hanteras enligt Personuppgiftslagen (PuL: 1998:204). Ansvarig för dina personuppgifter är Umeå universitet.

Frivillighet.
Din medverkan bygger på frivillighet. Du kan av den anledningen när som helst avbryta ditt deltagande utan att detta påverkar ditt framtida omhändertagande.

Kontaktperson:
Carola Jonasson, Institutionen för Integrativ medicinsk biologi, Umeå Universitet
carola.jonasson@physiol.umu.se Tel: 072-208 75 58

UMEÅ UNIVERSITET
Institutionen för Integrativ medicinsk biologi

INFORMERAT SAMTYCKE

Datum:………………………………….

Jag (för och efternamn textat)……………………………………………………………………………………………………
har blivit muntligen och skriftligen informerad om projektet och samtycker till att delta i projektet. Jag känner till att mitt deltagande är helt frivilligt, samt att jag när som helst och utan närmare förklaring kan avbryta mitt deltagande utan att detta påverkar mitt framtida omhändertagande.

Personnummer:……………………………..…………..

Man □
Kvinna □
Höger dominant □

……..

Namnteckning

Försöksansvarig: Benoni Edin, prof. Tel 090-786 54 98
Fysiologi, IMB, Umeå Universitet, 901 87 Umeå

Links to suppliers of equipment used

FastTrak Motion Tracking. Polhemus.
http://www.polhemus.com/?page=motion_fastrak

F/T Data Acquisition Six-Axis Force/Torque Sensor System. ATI Industrial Automation.
http://www.ati-ia.com/app_content/documents/9610-05-1017%20DAQ.pdf

ActiCAP. Brain Products GmbH.
http://www.brainproducts.com/productdetails.php?id=4&tab=1

BrainAmp Standard. Brain Products GmbH.
http://www.brainproducts.com/productdetails.php?id=1

BrainVision Recorder. Brain Products GmbH.
http://www.brainproducts.com/downloads.php?kid=2

Series schedule for each participant

Experimental series:

Series:	*Practice*	#1	#2	#3	#4	#5	#6	#7	#8	#9	#10
**Participant 1**		*Weight 1*	*Surface 1*	*Mixed 1*	*Weight 2*	*Mixed 2*	*Surface 2*	*Weight 3*	*Weight 4*	*Weight 5*	***Weight 6***
**Participant 2**	-	*Mixed 3*	*Surface 3*	*Weight 3*	*Surface 1*	*Weight 1*	*Mixed 1*	*Weight 2*	***Weight 6***	*Weight 4*	*Weight 5*
**Participant 3**	-	*Weight 1*	*Mixed 1*	*Surface 1*	*Mixed 2*	*Surface 2*	*Weight 2*	*Weight 3*	*Weight 4*	*Weight 5*	***Weight 6***
**Participant 4**	-	*Surface 2*	*Mixed 2*	*Weight 2*	*Mixed 3*	*Weight 3*	*Surface 3*	*Weight 1*	*Weight 5*	***Weight 6***	*Weight 4*
**Participant 5**	-	*Mixed 3*	*Weight 3*	*Surface 3*	*Weight 1*	*Surface 1*	*Mixed 1*	*Weight 2*	***Weight 6***	*Weight 4*	*Weight 5*
**Participant 6**	-	*Weight 1*	*Surface 1*	*Mixed 1*	*Weight 2*	*Mixed 2*	*Surface 2*	*Weight 3*	*Weight 4*	*Weight 5*	***Weight 6***
**Participant 7**	-	*Surface 2*	*Weight 2*	*Mixed 2*	*Surface 3*	*Mixed 3*	*Weight 3*	*Weight 1*	*Weight 5*	***Weight 6***	*Weight 4*
**Participant 8**	-	*Mixed 3*	*Surface 3*	*Weight 3*	*Surface 1*	*Weight 1*	*Mixed 1*	*Weight 2*	***Weight 6***	*Weight 4*	*Weight 5*
**Participant 9**	-	*Weight 1*	*Mixed 1*	*Surface 1*	*Mixed 2*	*Surface 2*	*Weight 2*	*Weight 3*	*Weight 4*	*Weight 5*	***Weight 6***
**Participant 10**	-	*Surface 2*	*Mixed 2*	*Weight 2*	*Mixed 3*	*Weight 3*	*Surface 3*	*Weight 1*	*Weight 5*	***Weight 6***	*Weight 4*
**Participant 11**	-	*Mixed 3*	*Weight 3*	*Surface 3*	*Weight 1*	*Surface 1*	*Mixed 1*	*Weight 2*	***Weight 6***	*Weight 4*	*Weight 5*
**Participant 12**	-	*Weight 1*	*Surface 1*	*Mixed 1*	*Weight 2*	*Mixed 2*	*Surface 2*	*Weight 3*	*Weight 4*	*Weight 5*	***Weight 6***

Representation in the data structures:

Only EEG data is available on figshare for series 'Weight 6' and this is found in files **HS_P1_ST.mat – HS_P12_ST.mat**

Series:	S1	S2	S3	S4	S5	S6	S7	S8	S9	ST (S10)
**Participant 1**	*Weight 1*	*Surface 1*	*Mixed 1*	*Weight 2*	*Mixed 2*	*Surface 2*	*Weight 3*	*Weight 4*	*Weight 5*	*Weight 6*
**Participant 2**	*Mixed 3*	*Surface 3*	*Weight 3*	*Surface 1*	*Weight 1*	*Mixed 1*	*Weight 2*	*Weight 4*	*Weight 5*	*Weight 6*
**Participant 3**	*Weight 1*	*Mixed 1*	*Surface 1*	*Mixed 2*	*Surface 2*	*Weight 2*	*Weight 3*	*Weight 4*	*Weight 5*	*Weight 6*
**Participant 4**	*Surface 2*	*Mixed 2*	*Weight 2*	*Mixed 3*	*Weight 3*	*Surface 3*	*Weight 1*	*Weight 5*	*Weight 4*	*Weight 6*
**Participant 5**	*Mixed 3*	*Weight 3*	*Surface 3*	*Weight 1*	*Surface 1*	*Mixed 1*	*Weight 2*	*Weight 4*	*Weight 5*	*Weight 6*
**Participant 6**	*Weight 1*	*Surface 1*	*Mixed 1*	*Weight 2*	*Mixed 2*	*Surface 2*	*Weight 3*	*Weight 4*	*Weight 5*	*Weight 6*
**Participant 7**	*Surface 2*	*Weight 2*	*Mixed 2*	*Surface 3*	*Mixed 3*	*Weight 3*	*Weight 1*	*Weight 5*	*Weight 4*	*Weight 6*
**Participant 8**	*Mixed 3*	*Surface 3*	*Weight 3*	*Surface 1*	*Weight 1*	*Mixed 1*	*Weight 2*	*Weight 4*	*Weight 5*	*Weight 6*
**Participant 9**	*Weight 1*	*Mixed 1*	*Surface 1*	*Mixed 2*	*Surface 2*	*Weight 2*	*Weight 3*	*Weight 4*	*Weight 5*	*Weight 6*
**Participant 10**	*Surface 2*	*Mixed 2*	*Weight 2*	*Mixed 3*	*Weight 3*	*Surface 3*	*Weight 1*	*Weight 5*	*Weight 4*	*Weight 6*
**Participant 11**	*Mixed 3*	*Weight 3*	*Surface 3*	*Weight 1*	*Surface 1*	*Mixed 1*	*Weight 2*	*Weight 4*	*Weight 5*	*Weight 6*
**Participant 12**	*Weight 1*	*Surface 1*	*Mixed 1*	*Weight 2*	*Mixed 2*	*Surface 2*	*Weight 3*	*Weight 4*	*Weight 5*	*Weight 6*

Information about participants

Participant Number	Gender	Number of series	Number of trials	Laterality index*	Notes
1	F	10	328	70	Not naïve to study's purpose.
2	F	10	328	—	Late reaction time to the light events. Slow movements.
3	M	10	328	80	Weak signal on EMG-3 (Flexor Digitorum)
4	F	10	328	80	EEG gel was added before the second series.
5	M	10	328	95	Weak signals on EMG-3 (Flexor Digitorum) and EMG-5 (First Dorsal Interosseus).
6	F	10	328	90	Early problems with EEG. An hour delay due to changing electrodes, changing batteries and adding gel. Noise from Polhemus on EMG-5 (First Dorsal Interosseus).
7	F	10	328	95	None.
8	M	10	328	65	5 years capoeira training. Unusually quick EEG relaxation. Some unexpected breaks in EMG-1 (Anterior Deltoid) and EMG-5 (First Dorsal Interosseus).
9	F	10	328	95	Sometimes makes small movements with left hand and foot. A few unexpected breaks in EMG-2 (Brachioradialis).
10	F	10	328	80	Rare big spikes in EMG-4 (Common Extensor Digitorum) and short sequences of flat lines in EMG-1 (Anterior Deltoid).
11	F	10	328	60	None
12	M	10	328	80	Rare big spikes in EMG-4 (Common Extensor Digitorum) and brief periods of noise in EMG-3 (Flexor Digitorum).

*) The *Laterality Index* was obtained from http://www.brainmapping.org/shared/Edinburgh.php# which is based on the Edinburg inventory: Oldfield RC. "The assessment and analysis of handedness: the Edinburgh inventory." Neuropsychologia 1971, 9(1):97-113.

In the 11 participants for which this index was calculated, the lowest value corresponds to the "3rd decile right", the highest value the "9th decile right".

Check-list for instructions to participants

1. **Give information regarding the aim of the study:**
   - *The aim is to study how the brain and muscles are coordinated when handling an object.*

2. **Information regarding the registration:**
   - *We register EEG from the brain. It is very important that you sit as still as possibly. Avoid additional muscle activity. Don´t talk during the performance of the task. You will be wearing earplugs. Focus on the task.*
   - *EMG will be registered from muscles on the right arm.*
   - *We will register where the hand, thumb and index finger are during the performance of the task.*
   - *There are no known risks with the experiments.*

3. **Describe the task**
   - *Sit close to the table, relax your shoulder and place your upper arm next to your body. The elbow joint shall be higher than the wrist. During performance of the task, the forearm shall not touch the table. Your left arm should rest close to your waist.*
   - *The red light is the signal to reach out and lift the object. Grasp the object with your thumb and index finger, in the middle of the grey surface and lift the object about 5 cm from the table.*
   - *You should lift the object into the circle and hold it there until the red light turns off.*
   - *Place the object on the table and place your arm next to your body. You shall rest your hand on the "blue surface" – relax your shoulder.*
   - *Before we start the registration you will get to practice the task a few times.*

4. **Risk, reimbursements and voluntary participation**
   - *You will be paid 100 SEK for each hour and the whole experiment will take about 3 hours and 15 minutes (~45 min preparation + 2.5 hours of performing the task)*
   - *The participation is voluntary and you may quit at any time.*
   - *If you have any questions, feel free to ask before we start (or between the tasks).*

5. **Consent form**

Ensure that the participant signs the consent form

Försöksansvarig: Benoni Edin, prof. Tel 090-786 54 98
Fysiologi, IMB, Umeå Universitet, 901 87 Umeå
