# Supplementary figures and images for: Multi-channel EEG recordings during 3,936 grasp and lift trials with varying weight and friction
Source: Sci Data. 2014 Nov 25;1:140047. doi: 10.1038/sdata.2014.47 (PMC4365902; doi:10.1038/sdata.2014.47)

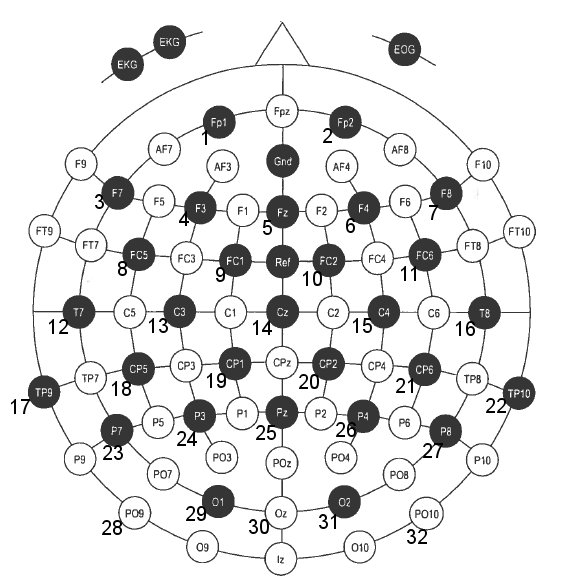

Supplement: Supplementary File 2—Utilities.zip [file sdata201447-s2.zip › Supplementary File 2 - Utilities/Utilities/EEG_Electrode_Numbering.jpg]
